# Stellenwert der Immuntherapie in der perioperativen Behandlung des lokalisierten muskelinvasiven Harnblasenkarzinoms

**DOI:** 10.1007/s00120-022-01983-5

**Published:** 2022-11-30

**Authors:** J. Lewerich, S. C. Schmid, J. E. Gschwend, M. Retz

**Affiliations:** 1 Klinik und Poliklinik für Urologie, Klinikum rechts der Isar der Technischen Universität München, Ismaninger Str. 22, 81675 München, Deutschland; 2 Interdisziplinare Arbeitsgruppe BlasenCarcinom (IABC) der DKG e. V., Deutsche Krebsgesellschaft, Berlin, Deutschland

**Keywords:** Urothelkarzinom, Nivolumab, Chemotherapie, Immun-Checkpoint-Inhibitoren, Harnblasenkarzinom, Bladder cancer, Perioperative therapy, Chemotherapy, Immune checkpoint inhibitors, Radical cystectomy

## Abstract

Immun-Checkpoint-Inhibitoren (ICI) sind als Erst- und Zweitlinientherapien beim metastasierten bzw. lokal fortgeschrittenen Urothelkarzinom als Standard etabliert. Der perioperative Einsatz der Immuntherapie steht nun im Fokus aktueller Entwicklungen und Forschung in der Uroonkologie. Aktuelle Studien untersuchen ICI als Monotherapie oder in Kombination mit Chemotherapie oder Bestrahlung im perioperativen Setting. Dieser Artikel soll einen Überblick über die neuen Studienkonzepte, die aktuell publizierten Daten und daraus entstehenden Therapiekonzepte sowie einen Ausblick auf zukünftige Entwicklungen bieten.

## Abstract (en)

Immune checkpoint inhibitors are standard of care in the treatment of metastatic and locally advanced urothelial cancer. Their use in perioperative treatment is currently under investigation as monotherapy as well as in combination with chemotherapy or radiation regimens. This article provides an overview of recent trials, current data as well as an outlook on future developments in the perioperative management of urothelial cancer.

Immun-Checkpoint-Inhibitoren (ICI) haben einen festen Stellenwert in der palliativen Systemtherapie des metastasierten oder lokal fortgeschrittenen Urothelkarzinoms. Aktuelle Studien untersuchen Checkpoint-Inhibitoren auch in der perioperativen Systemtherapie um die radikale Zystektomie in kurativer Intention.

Die Standardtherapie des muskelinvasiven Urothelkarzinoms der Harnblase ist die radikale Zystektomie mit pelviner Lymphadenektomie. Die Langzeitprognose wird vom Tumorstadium bestimmt. Während sich bei Patienten mit einem organbegrenzten Befall (pT2) eine 5‑Jahres-Überlebensrate von 72 % zeigt, ist die Prognose bei lokal fortgeschrittenem Befund erheblich schlechter. Bei Infiltration des perivesikalen Fettgewebes (pT3) fällt die Gesamtüberlebensrate nach 5 Jahren auf 43 %, bei Infiltration von Nachbarorganen (pT4) auf 28 %. Besonders schlecht ist die Prognose bei Feststellung einer Lymphknotenmetastasierung, in diesem Fall ist mit einer 5‑Jahres-Überlebensrate von ca. 20 % zu rechnen [10]. Der unzureichende Therapieeffekt einer alleinigen chirurgischen Behandlung bedingt die Notwendigkeit einer perioperativen Systemtherapie.

## Aktueller Standard

Entsprechend der S3-Leitlinie Harnblasenkarzinom soll jedem Patienten mit einem muskelinvasiven Harnblasenkarzinom (pT2-pT4a pN0) eine neoadjuvante Systemtherapie mit einer Cisplatin-basierten Polychemotherapie angeboten werden. Bei Patienten nach alleiniger Zystektomie und postoperativem Nachweis eines lokal fortgeschrittenen Blasentumors (≥ pT3) oder pelvinen Lymphknotenbefalls (pN+) ist eine adjuvante Cisplatin-basierte Chemotherapie indiziert [19]. Zusätzlich weist die S3-Leitlinie ‚Blasenkarzinom‘ darauf hin, dass der Überlebensvorteil der perioperativen Systemtherapie nur mit einer Cisplatin-basierten Polychemotherapie erreicht werden kann. Zum perioperativen Einsatz einer Carboplatin-haltigen Systemtherapie liegen keine prospektiven Daten vor, zumal die Effektivität von Carboplatin im metastasierten Stadium deutlich geringer ausfällt. Aufgrund häufiger Kontraindikationen sind jedoch viele Patienten für eine perioperative Chemotherapie ungeeignet. Laut einer Arbeit von Galsky et al. sind hiervon nahezu 50 % der Patienten betroffen, vorwiegend aufgrund von Nierenfunktionsstörungen [8].

Die Etablierung der ICI im metastasierten und fortgeschrittenen Erkrankungsstadium führte unweigerlich zur Initiierung zahlreicher Studien, in denen die Immuntherapie im perioperativen Einsatz als Monosubstanz oder in Kombination mit der Chemotherapie untersucht wurde ([2–4, 18, 21]; Tab. 1).

**Table 1:** 

Studie	*n*	Therapie	Endpunkte	Ergebnis
*NCT02736266* PURE-01	114	3 × Pembrolizumab 200 mg (q3w) vor radikaler Zystektomie	**ypT0 ypN0-Rate**	37 % (95 %-KI: 28–46)
*NCT02662309* ABACUS	95	2 × Atezolizumab 1200 mg (q3w) vor radikaler Zystektomie	**ypT0 ypN0-Rate** *Subgruppe PD-L1 +*	31 % (95 %-KI: 21–41) *37* *% (95* *%-KI: 21–55)*
*NCT03387761* NABUCCO Kohorte 1	24	2 × Ipilimumab 3 mg/kg (q3w) + 1 × Nivolumab 1 mg/kg + 1 × Nivolumab 3 mg/kg vor radikaler Resektion	**OP in ≤** **12 Wochen** *Subgruppe ypT0 ypN0*	96 % (95 %-KI: 79–100) *46* *% (95* *%-KI: 26–67)*
*NCT03387761* NABUCCO Kohorte 2	30	2 × Ipilimumab 3 mg/kg + 2 × Nivolumab 1 mg/kg + 1 × Nivolumab 3 mg/kg (q3w) vor radikaler Resektion *vs.* 2 × Ipilimumab 1 mg/kg + 3 × Nivolumab 3 mg/kg (q3w) vor radikaler Resektion	**ypT0 ypN0-Rate**	43 % *(95* *%-KI: keine Angabe)* 7 % *(95* *%-KI: keine Angabe)*

Primärer Endpunkt **fett** markiert

*95* *%-KI* 95 %-Konfidenzintervall, *PD-L1* „programmed cell death ligand 1“

## Immuntherapie im neoadjuvanten Ansatz vor radikaler Zystektomie

### Checkpoint-Inhibitoren als Monotherapie

Es liegen drei prospektive Studien zum Einsatz von Checkpoint-Inhibitoren als neoadjuvante Systemtherapie vor radikaler Zystektomie vor (Tab. 1).

In der Phase-II-Studie PURE-01 (NCT02736266) untersuchten Necchi et al. den Einsatz von 3 Zyklen Pembrolizumab 200 mg im 3‑Wochen-Intervall mit anschließender radikaler Zystektomie bei 50 Patienten mit einem muskelinvasiven Urothelkarzinom ohne Anhalt für eine lymphogene oder viszerale Metastasierung (cT2-3b cN0 cM0). Die Zystektomie erfolgte mindestens eine bis maximal 3 Wochen nach der letzten Gabe von Pembrolizumab. Als primärer Endpunkt wurde die Rate an pathologischen Komplettremissionen (pT0 pN0) gewählt. Bei 42 % der Patienten (95 %-Konfidenzintervall [95 %-KI]: 28,2–56,8) wurde nach abgeschlossener Therapie ein pT0-Befund festgestellt. Unter den PD-L1-positiven Tumoren erhöhte sich der Anteil auf 54 % [13]. Die Einschlusskriterien wurden im Studienprotokoll erweitert, so dass auch Patienten mit einer nicht dominant urothelialen Differenzierung sowie mit einem cT4a-Stadium eingeschlossen werden konnten. In der aktualisierten Analyse mit zusätzlich 64 eingeschlossenen Studienkandidaten zeigte die Gesamtpopulation eine ypT0-Rate von 37 % (95 %-KI: 28–46) und die ≤ ypT1-Rate lag bei 55 % (95 %-KI: 46–65) [14].

In der 2. prospektiven Phase-II-Studie ABACUS (NCT02662309) untersuchten Powles et al. den Einsatz des PD-L1-Liganden („programmed cell death ligand 1“) Atezolizumab. Eingeschlossen wurden 95 Patienten mit einem muskelinvasiven Urothelkarzinom der Harnblase (cT2-4a cN0 cM0). Alle Patienten wurden mit 2 Zyklen Atezolizumab 1200 mg im 3‑Wochen-Intervall gefolgt von einer radikalen Zystektomie behandelt. Der primäre Endpunkt war die Rate an pathologischen Komplettremissionen (pT0 pN0). Von den 95 Patienten erhielten 87 (92 %) eine radikale Zystektomie. Unter den 8 nicht operierten Patienten zeigten sich 2 Todesfälle, von denen einer als therapiebedingt bewertet wurde. Weitere 3 Patienten konnten aufgrund einer Verschlechterung des klinischen Allgemeinzustands nicht operiert werden. Ein Patient hatte unter der Immuntherapie bereits eine Tumorprogression, ein Patient lehnte die chirurgische Therapie ab. Ein weiterer Patient zog seine Einwilligung zur Studienteilnahme präoperativ zurück. Insgesamt zeigten 31 % (95 %-KI: 21–41) der Patienten einen ypT0-Befund, in der Subgruppe mit einem PD-L1-positiven Tumorstatus lag die ypT0-Rate bei 37 % (95 %-KI: 21–55; [15, 22]).

In der 3. Phase‑I Studie NABUCCO (NCT03387761) untersuchten van Dijk et al. den Einsatz einer kombinierten Immuntherapie aus Ipilimumab und Nivolumab bei 54 Patienten mit einem lokal fortgeschrittenen oder primär lymphogen metastasierten Urothelkarzinoms der Harnblase oder des oberen Harntrakts (cT3-4a cN0 cM0 oder cT1-4a cN+cM0). Die Patienten wurden in 2 Kohorten aufgeteilt. In der Kohorte 1 wurde Ipilimumab an Tag 1 und 22 in einer Dosis von 3 mg/kg, Nivolumab an Tag 22 mit 1 mg/kg sowie an Tag 43 mit 3 mg/kg verabreicht. Der primäre Endpunkt war die Rate an Patienten, bei denen innerhalb von 9–12 Wochen nach Beginn der Systemtherapie die radikale Resektion erfolgte. Dieser wurde bei 96 % der Patienten (95%-KI: 79–100) erreicht. Als sekundärer Endpunkt wurde die Rate an pathologischen Komplettremissionen ausgewertet, diese betrug 46 % (95 %-KI: 26–67; [23]).

In der Kohorte 2 wurden 30 Patienten im Verhältnis 1:1 in 2 Arme randomisiert. Beide Arme erhielten an Tag 1 und 22 Nivolumab und Ipilimumab, in Arm A wurde Ipilimumab mit 3 mg/kg und Nivolumab mit 1 mg/kg, in Arm B wurde Ipilimumab mit 1 mg/kg und Nivolumab mit 3 mg/kg verabreicht. An Tag 43 erhielten die Patienten in beiden Armen Nivolumab mit 3 mg/kg. Die radikale Resektion erfolgte 9–12 Wochen nach Beginn der Systemtherapie. Die Ergebnisse wurden 2021 auf dem ESMO-Kongress (European Society of Medical Oncology) präsentiert, die Rate an pathologischen Komplettremissionen betrug in Arm A 43 % (*n* = 6) und in Arm B 7 % (*n* = 1). Der Anteil an partiellen Remissionen zu einem nicht muskelinvasiven Tumorstadium (≤ ypT1) betrug in Arm A 57 % (*n* = 8) sowie in Arm B 29 % (*n* = 4; [24]).

Zusammenfassend lag die Rate an pathologischen Komplettremissionen bei Blasenkarzinompatienten mit neoadjuvanter Checkpoint-Inhibitor-Therapie gefolgt von radikaler Zystektomie zwischen 31 % und 46 %, in der Subgruppe mit einem PD-L1-positiven Tumorstatus zwischen 37 % und 54 %.

### Kombinierte Immunchemotherapie

Eine weitere naheliegende Option stellt die Hinzunahme von Checkpoint-Inhibitoren in etablierte Chemotherapieregime dar. Zum Einsatz kommen sowohl rein neoadjuvante Therapiekonzepte als auch sog. „Sandwich“-Regime, bei denen zusätzlich zur neoadjuvanten Immunchemotherapie eine adjuvante Immuntherapie nach der radikalen Zystektomie angeschlossen wird. Es liegen Daten aus vier Phase-II-Studien zum neoadjuvanten Einsatz vor (Tab. 2).

**Table 2:** 

Studie	*n*	Therapie	Endpunkte	Ergebnis
*NCT02989584*	44	4 × Gemcitabin/Cisplatin + 6 × Atezolizumab 1200 mg (q3w) vor radikaler Zystektomie	**<** **ypT2 ypN0-Rate** *Subgruppe ypT0 ypN0*	69 % (95 %-KI: keine Angabe) *41* *% (95* *%-KI: keine Angabe)*
*NCT03294304* BLASST‑1	41	4 × Gemcitabin/Cisplatin + 4 × Nivolumab 360 mg (q3w) vor radikaler Zystektomie	**≤** **ypT1 ypN0-Rate** *Subgruppe ypT0/Tis ypN0*	66 % (95 %-KI: keine Angabe) *49* *% (95* *%-KI: keine Angabe)*
*NCT03674424* AURA Kohorte 1	110	4 × Methotrexat/Vinblastin/Doxorubicin/Cisplatin (q2w) + Avelumab 10 mg/kg (q2w) vor radikaler Zystektomie *vs.* 4 × Gemcitabin/Cisplatin (q3w) + Avelumab 10 mg/kg (q2w) vor radikaler Zystektomie	**ypT0/Tis ypN0-Rate**	Rekrutierung abgeschlossen
*NCT03674424* AURA Kohorte 2	56	4 × Gemcitabin/Paclitaxel (q3w) + Avelumab 10 mg/kg (q2w) vor radikaler Zystektomie *vs.* 4 × Avelumab 10 mg/kg (q2w)	**ypT0/Tis ypN0-Rate**	18 % (95 %-KI: 6–37) 36 % (95 %-KI: 19–56)
*NCT03406650* SAKK 06/17	61	4 × Gemcitabin/Cisplatin + Durvalumab 1500 mg (q3w) vor radikaler Resektion plus Adjuvanz 10 × Durvalumab 1500 mg (q4w)	**EFS nach 2 Jahren** ypT0 ypN0-Rate ≤ ypT1 ypN0-Rate	76,1 % (95 %-KI: 62,3–85,3) 34 % (95 %-KI: 21,5–48,3) 60,4 % (95 %-KI: 46,0–73,5)

Primärer Endpunkt **fett** markiert

*95* *%-KI* 95 %-Konfidenzintervall, *EFS* ereignisfreies Überleben

In der 1. Phase-II-Studie (NCT02989584) schlossen Funt et al. insgesamt 44 Patienten mit einem muskelinvasiven Blasenkarzinom (cT2-4a cN0 cM0) ein, von denen 39 in die Auswertung einflossen. Die neoadjuvante Therapie beinhaltete eine Immuntherapie aus 6 Gaben Atezolizumab kombiniert mit 4 Zyklen Gemcitabin/Cisplatin (Gem/Cis)-Chemotherapie im 3‑Wochen-Intervall. Primärer Endpunkt war die pathologische Ansprechrate definiert als ≤ pT2 pN0. Die pathologische Ansprechrate (≤ ypT2 ypN0) lag bei 69 %, die ypT0-Rate als sekundärer Endpunkt bei 41 %. Die PD-L1-Expression war in der Studienpopulation mit 10 % gering, sodass keine Subgruppenanalyse erfolgen konnte [7].

In der 2. Phase-II-Studie BLASST‑1 (NCT03294304) von Gupta et al. wurden 41 Patienten mit einem muskelinvasiven Blasenkarzinom (cT2-4a cN0/1 cM0) eingeschlossen. Es wurde eine neoadjuvante Immunchemotherapie aus 4 Gaben Nivolumab und 4 Zyklen Gem/Cis-Chemotherapie im 3‑Wochen-Intervall durchgeführt. Die radikale Zystektomie erfolgte innerhalb von 8 Wochen nach Abschluss der Systemtherapie. Die pathologische Ansprechrate mit ≤ ypT1 lag bei 66 %. Der Anteil pathologischer Komplettremissionen, definiert als ypT0/Tis, lag bei 49 % [9].

In der 3. Phase-II-Studie AURA (NCT03674424) untersuchten Chanza et al. bei 166 Patienten mit einem muskelinvasiven Blasenkarzinom (cT2-4a cN0-2 cM0) den Einsatz des PD-L1-Inhibitors Avelumab. Die Patienten wurden je nach Eignung für eine Cisplatin-haltige Chemotherapie in 2 Kohorten aufgeteilt.

In Kohorte 1 wurden 110 Cisplatin-fitte Patienten eingeschlossen, die im Verhältnis 1:1 in 2 Therapiearme randomisiert wurden. Im 1. Arm wurden 4 Zyklen Chemotherapie mit Methotrexat/Vinblastin/Doxorubicin/Cisplatin (MVAC) kombiniert mit Avelumab im 2‑Wochen-Intervall verabreicht. Im 2. Arm erhielten Patienten 4 Zyklen Chemotherapie mit Gem/Cis im 3‑Wochen-Intervall sowie parallel Avelumab im 2‑Wochen-Intervall. Die radikale Zystektomie erfolgte jeweils 3–6 Wochen nach Abschluss der Systemtherapie. Die Ergebnisse dieser Kohorte liegen noch nicht vor.

In Kohorte 2 wurden 56 Cisplatin-ungeeignete Patienten eingeschlossen, die im Verhältnis 1:1 in 2 Therapiearme randomisiert wurden. In Arm A wurde eine Immunchemotherapie mit 4 Zyklen Gemcitabin/Paclitaxel im 3‑Wochen-Intervall sowie parallel Avelumab im 2‑Wochen-Intervall verabreicht. Anschließend erfolgte die radikale Zystektomie 3–6 Wochen nach Abschluss der Systemtherapie. In Arm B wurde eine Monotherapie mit 4 Zyklen Avelumab im 2‑Wochen-Intervall durchgeführt. Die radikale Zystektomie erfolgte innerhalb von 2 Wochen nach der letzten Gabe der Immuntherapie. Primärer Endpunkt war die Rate an pathologische Komplettremissionen, definiert als ypT0/Tis. Erste Ergebnisse dieser Kohorte wurden 2022 auf dem ASCO-Meeting (American Society of Clinical Oncology) präsentiert. Im Arm A betrug die Rate an pathologischen Komplettremissionen 18 % (95 %-KI: 6–37), in Arm B dagegen 36 % (95 %-KI: 19–56; [12]).

In der 4. Phase-II-Studie SAKK 06/17 (NCT03406650) untersuchten Cathomas et al. bei 61 Patienten mit einem muskelinvasiven Urothelkarzinom des unteren und oberen Harntrakts (cT2-4a cN0/1 cM0) den Einsatz des PD-L1-Inhibitors Durvalumab. Patienten erhielten zunächst 4 Zyklen Gem/Cis-Chemotherapie kombiniert mit Durvalumab im 3‑Wochen-Intervall mit anschließender radikal chirurgischer Resektion. Anschließend wurden bis zu 10 Zyklen einer adjuvanten Immuntherapie mit Durvalumab im 4‑Wochen-Intervall verabreicht. Der primäre Endpunkt war das ereignisfreie Überleben (EFS) 2 Jahre nach Therapiebeginn, das pathologische Ansprechen wurde als sekundärer Endpunkt ausgewertet. Erste Resultate wurden auf dem ASCO-Meeting 2022 präsentiert. Die radikale Resektion erfolgte bei 53 von 58 auswertbaren Patienten, davon 52 radikale Zystektomien. Die EFS-Rate nach 2 Jahren betrug 76,1 % (95 %-KI: 62,3–85,3). Die Rate an pathologischen Komplettremissionen (ypT0) betrug 34 % (95 %-KI: 21,5–48,3), eine partielle Remission zu einem nicht-muskelinvasiven Karzinom (≤ ypT1) wurde insgesamt bei 60,4 % (95 %-KI: 46,0–73,5) der Fälle erreicht [6].

Nach den vorliegenden Studiendaten scheint die neoadjuvante, Cisplatin-haltige Immunchemotherapie eine höhere Wirksamkeit im Vergleich zur Monoimmuntherapie zu erreichen. Der Einsatz von Paclitaxel bei Cisplatin-unfitten Patienten hingegen ergab ein schlechteres Ansprechen als die alleinige Immuntherapie. Eine noch höhere Effektivität verspricht man sich durch ein „Sandwich“-Regime um die radikale Zystektomie. In diesem Studiendesign wird zunächst eine neoadjuvante Immunchemotherapie und radikale Zystektomie durchgeführt, gefolgt von einer zeitlich begrenzten adjuvanten Monoimmuntherapie. Die ersten Studienergebnisse werden frühestens für 2023/24 erwartet (Tab. 3 und 4).

**Table 3:** 

Studie	*n*	Therapie	Primärer Endpunkt	Status
*NCT03661320* ENERGIZE	861	4 × Gemcitabin/Cisplatin + Nivolumab 360 mg (q3w) vor radikaler Zystektomie plus Adjuvanz 9 × Nivolumab 480 mg (q4w) *vs.* 4 × Gemcitabin/Cisplatin vor radikaler Zystektomie plus Nachbeobachtung	pCR-Rate EFS	Aktiv
*NCT03924856* KEYNOTE-866	870	4 × Gemcitabin/Cisplatin + Pembrolizumab 200 mg (q3w) vor radikaler Zystektomie plus Adjuvanz 13 × Pembrolizumab 200 mg (q3w) *vs.* 4 × Gemcitabin/Cisplatin + Placebo vor radikaler Zystektomie plus Adjuvanz 13x Placebo (q3w)	pCR-Rate EFS	Aktiv
*NCT03732677* NIAGARA	988	4 × Gemcitabin/Cisplatin + Durvalumab 1500 mg (q3w) vor radikaler Zystektomie plus Adjuvanz 8 × Durvalumab 1500 mg (q4w) *vs.* 4 × Gemcitabin/Cisplatin vor radikaler Zystektomie plus Nachbeobachtung	pCR-Rate EFS	Abgeschlossen Nachbeobachtung
*NCT04700124* KEYNOTE-B15/ EV304	784	4 × Enfortumab-Vedotin + Pembrolizumab 200 mg (q3w) vor radikaler Zystektomie plus Adjuvanz 5 × Enfortumab-Vedotin +13 × Pembrolizumab 200 mg (q3w) *vs.* 4 × Gemcitabin/Cisplatin vor radikaler Zystektomie plus Nachbeobachtung	pCR-Rate EFS	Aktiv

*EFS* ereignisfreies Überleben, *pCR* pathologische Komplettremission

**Table 4:** 

Studie	*n*	Therapie	Primärer Endpunkt	Status
*NCT04960709* VOLGA	830	3 × Durvalumab 1500 mg + Enfortumab-Vedotin 1,25 mg/kg + Tremelimumab 75 mg (q3w) vor radikaler Zystektomie plus Adjuvanz 1 × Tremelimumab 75 mg + 9 × Durvalumab 1500 mg (q4w) *vs.* 3 × Durvalumab 1500 mg + Enfortumab-Vedotin 1,25 mg/kg (q3w) vor radikaler Zystektomie plus Adjuvanz: 9 × Durvalumab 1500 mg (q4w) *vs.* radikale Zystektomie plus Nachbeobachtung	Sicherheit Verträglichkeit pCR-Rate EFS	Aktiv
*NCT03924895* KEYNOTE-905/ EV303	857	3 × Pembrolizumab 200 mg + Enfortumab-Vedotin 1,25 mg/kg (q3w) vor radikaler Zystektomie plus Adjuvanz 6 × Enfortumab-Vedotin 1,25 mg/kg + 14 × Pembrolizumab 200 mg (q3w) *vs.* 3 × Pembrolizumab 200 mg (q3w) vor radikaler Zystektomie plus Adjuvanz 14 × Pembrolizumab 200 mg (q3w) *vs.* radikale Zystektomie plus Nachbeobachtung	pCR-Rate EFS	Aktiv

*EFS* ereignisfreies Überleben

Für die Cisplatin-geeignete Patientengruppe können vier wichtige Studien in Deutschland angeboten werden: Die randomisierte Phase-III-Studie ENERGIZE (NCT03661320) untersucht die klassische neoadjuvante Gem/Cis-Chemotherapie im Vergleich zur neoadjuvanten Immunchemotherapie mit Nivolumab und Gem/Cis gefolgt von radikaler Zystektomie sowie adjuvanter Immuntherapie mit Nivolumab für 9 Monate. Analog wird in der Phase-III-Studie KEYNOTE-866 (NCT03924856) im experimentellen Arm eine neoadjuvante Immunchemotherapie mit Pembrolizumab und Gem/Cis mit anschließender radikaler Zystektomie, gefolgt von Pembrolizumab für 9 Monate überprüft. Das nahezu gleiche Studienkonzept wird in der Phase-III-Studie NIAGARA (NCT03732677) mit dem Checkpoint-Inhibitor Durvalumab verfolgt. In der Studie EV304/KEYNOTE-B15 (NCT04700124) wird im experimentellen Arm auf die Cisplatin-haltige Chemotherapie verzichtet: Es wird eine neue Kombination aus Pembrolizumab und dem zytotoxischen Antikörperkonjugat Enfortumab-Vedotin sowohl neoadjuvant als auch adjuvant um die radikale Zystektomie überprüft [11]. Enfortumab-Vedotin wurde bisher beim metastasierten bzw. lokal fortgeschrittenen Urothelkarzinom in der Drittlinie nach Progress unter Chemo- und Immuntherapie untersucht und konnte einen signifikanten Überlebensvorteil gegenüber einer Monochemotherapie mit Vinflunin, Paclitaxel bzw. Docetaxel zeigen [17].

Für die Cisplatin-unfähige Patientengruppe wird ebenfalls das „Sandwich“-Studiendesign angewendet, wobei hier überwiegend Mono- und Duoimmuntherapien in Kombination mit dem Antikörperkonjugat Enfortumab-Vedotin eingesetzt werden. Insgesamt können 2 wichtige Studien in Deutschland angeboten werden: Die dreiarmige randomisierte Phase-III-Studie VOLGA (NCT04960709) untersucht im Vergleich zur alleinigen radikalen Zystektomie im ersten Prüfarm eine neoadjuvante Kombination aus den Checkpoint-Inhibitoren Durvalumab und Tremelimumab plus Enfortumab-Vedotin, gefolgt von einer adjuvanten Duoimmuntherapie mit Durvalumab und Tremelimumab nach radikaler Zystektomie. Im 2. Prüfarm werden nach analogem Schema neoadjuvant Durvalumab plus Enfortumab-Vedotin und eine adjuvante Durvalumab-Monotherapie verabreicht. Die dreiarmige randomisierte Phase-III-Studie KEYNOTE-905/EV303 (NCT03924895) untersucht im Vergleich zur alleinigen Zystektomie im ersten Prüfarm Pembrolizumab und Enfortumab-Vedotin sowohl im neoadjuvanten als auch adjuvanten Ansatz; im 2. Prüfarm wird eine neoadjuvante und adjuvante Monotherapie mit Pembrolizumab durchgeführt.

### Kombinierte Radioimmuntherapie

Studiendaten von anderen Tumorentitäten zeigten synergistische Effekte von Immuntherapie und Bestrahlung als multimodales Konzept. Die Strahlentherapie kann eine vermehrte PD-L1-Expression auf Tumorzellen induzieren und somit die Immunantwort verstärken. Zudem sind sog. „Off-target“-Effekte beschrieben, bei denen die immunogene Wirkung der Bestrahlung auch ein Ansprechen von Tumorläsionen außerhalb des Bestrahlungsfeldes verursachen kann.

In der IIT-Studie RACE-IT (NCT03529890) untersuchten Schmid et al. den neoadjuvanten Einsatz einer Kombination aus 4 Zyklen Nivolumab 240 mg im 2‑Wochen-Intervall und lokaler Strahlentherapie der Harnblase und pelviner Lymphabflusswege mit 50,4 Gy über 28 Fraktionen und anschließender radikaler Zystektomie. Die Rekrutierung ist mittlerweile abgeschlossen und es konnten 33 Patienten mit einem lokal fortgeschrittenen Urothelkarzinom der Harnblase cT3-4a und/oder cN+ behandelt werden. Der primäre Endpunkt untersucht die Durchführbarkeit des multimodalen Therapiekonzepts bestehend aus neoadjuvanter Radioimmuntherapie und radikaler Zystektomie. Zu den sekundären Endpunkten gehören u. a. das pathologische Ansprechen, krankheitsfreies Überleben (DFS), das Gesamtüberleben sowie das Sicherheitsprofil. Erste Daten wurden auf dem ESMO-Kongress 2022 präsentiert, der primäre Endpunkt wurde bei 87,1% der Patienten erreicht. Die Rate an pathologischen Komplettremissionen betrug 38,7%, ein Downstaging zu einem nicht-muskelinvasiven Tumor konnte in 58,1% der Fälle erreicht werden [20].

## Immuntherapie als adjuvanter Ansatz nach radikaler Zystektomie

Ein hohes Rezidivrisiko besteht bei Blasentumorpatienten, die nach alleiniger radikaler Zystektomie ein lokal fortgeschrittenes Tumorstadium oder eine pelvine lymphogene Metastasierung (pT ≥ 3 und/oder pN+) aufweisen. Die S3-Leitlinie empfiehlt in dieser Situation eine adjuvante Cisplatin-basierte Polychemotherapie [19]. Allerdings findet sich eine Subgruppe an Patienten, die postoperativ nicht für Cisplatin geeignet sind und somit keine adjuvante Cisplatin-haltige Chemotherapie erhalten können. Für diese Hochrisikopatientengruppe gibt es keine S3-Leitlinienempfehlung. Eine weitere Problemgruppe sind Patienten, bei denen trotz neoadjuvanter Cisplatin-basierter Chemotherapie nach radikaler Zystektomie weiterhin ein muskelinvasives Tumorstadium und/oder pelvine Lymphknotenmetastasen (pT ≥ 2 und/oder pN+) nachweisbar sind. Aufgrund von fehlenden Studiendaten konnte die S3-Leitlinie auch für diese zweite Hochrisikogruppe keine Therapieempfehlung aussprechen. Aktuell gibt es 3 randomisierte Phase-III-Studien, die speziell für diese zwei Hochrisikopatientengruppen den adjuvanten Einsatz von Checkpoint-Inhibitoren überprüfen (Tab. 5).

**Table 5:** 

Studie	*n*	Therapie	Primärer Endpunkt	Ergebnis
*NCT03244384* AMBASSADOR	739	Chirurgische R0-Resektion plus Adjuvanz: Pembrolizumab 200 mg (q3w) vs. Beobachtung für 12 Monate	OS Gesamtpopulation DFS Gesamtpopulation	Rekrutierung abgeschlossen aktive Nachsorge
*NCT02450331* IMvigor010	809	Chirurgische R0-Resektion plus Adjuvanz: Atezolizumab 1200 mg (q3w) vs. Beobachtung für 12 Monate	DFS Gesamtpopulation	19,4 vs. 16,6 Mo. HR 0,89; *p* = 0,24
*NCT02632409* CHECKMATE-274	709	Chirurgische R0-Resektion plus Adjuvanz: Nivolumab 240 mg (q2w) vs. Placebo für 12 Monate	Median DFS Gesamtpopulation Median DFS PD-L1+ Subgruppe	20,8 vs. 10,8 Mo. HR 0,7; *p* < 0,001 Noch nicht erreicht HR 0,55; *p* < 0,001

*DFS* krankheitsfreies Überleben, *OS* Gesamtüberleben, *HR* Hazard Ratio, *PD-L1* „programmed cell death-ligand 1“, *Mo.* Monate, *OS* Gesamtüberleben

In der 1. Phase-III-Studie AMBASSADOR (NCT03244384) wurden zwei Patientengruppen eingeschlossen: Cisplatin-ungeeignete Patienten mit einem lokal fortgeschrittenen Tumorstadium und/oder pelviner lymphogener Metastasierung (pT ≥ 3 und/oder pN+) nach radikaler R0-Resektion eines muskelinvasiven Urothelkarzinoms der Harnblase oder des oberen Harntrakts. Die zweite Patientengruppe hatte bereits eine neoadjuvante Chemotherapie erhalten und zeigte nach radikal chirurgischer Operation weiterhin ein muskelinvasives Tumorstadium oder eine lymphogene Metastasierung (pT ≥ 2 und/oder pN+). Beide Gruppen wurden randomisiert und erhielten entweder eine adjuvante Therapie mit Pembrolizumab 200 mg im 3‑Wochen-Intervall für ein Jahr oder verblindet eine Placeboinfusion. Primäre Endpunkte der Studie sind das Gesamtüberleben und das krankheitsfreie Überleben. Die ersten Studiendaten werden für 2024 erwartet.

In der 2. randomisierten Phase-III-Studie IMvigor010 (NCT02450331) wurden die identischen Einschlusskriterien analog zur AMBASSADOR-Studie gewählt und es wurden insgesamt 809 Patienten eingeschlossen. Im adjuvanten Ansatz wurde der Checkpoint-Inhibitor Atezolizumab 1200 mg im 3‑Wochen-Intervall für ein Jahr im Vergleich zu einer Beobachtungsgruppe untersucht. Der primäre Endpunkt war das krankheitsfreie Überleben in der Gesamtpopulation. Nach einer medianen Nachbeobachtungszeit von 21,9 Monaten betrug das mediane krankheitsfreie Überleben im Prüfarm 19,4 Monate (95 %-KI: 15,9–24,8) und im Kontrollarm 16,6 Monate (95 %-KI: 11,2–24,8). Die HR für das krankheitsfreie Überleben betrug 0,89 (95 %-KI: 0,74–1,08). Es konnte kein statistisch signifikanter Vorteil für eine adjuvante Immuntherapie mit Atezolizumab gegenüber der Beobachtungsgruppe gezeigt werden (*p* = 0,24). In einem wissenschaftlichen Begleitprogramm konnte eine Subgruppe identifiziert werden, die bei Nachweis von zirkulierender Tumor-DNA (ctDNA) von der adjuvanten Immuntherapie mit Atezolizumab profitiert hatte. Patienten mit ctDNA-Nachweis zeigten mit adjuvanter Atezolizumab-Therapie im Vergleich zur Beobachtungsgruppe einen statistisch signifikanten Vorteil für das krankheitsfreie Überleben und das Gesamtüberleben. Aufgrund der ersten positiven wissenschaftlichen Studiendaten wurde die Phase-III-Studie IMvigor011 (NCT04660344) initiiert, die den Einsatz einer adjuvanten Immuntherapie mit Atezolizumab bei Patienten mit ctDNA-Nachweis nach erfolgter R0-radikaler Zystektomie untersucht [5, 16].

In der 3. Phase-III-Studie CHECKMATE-274 (NCT02632409) wurden ebenfalls die identischen Einschlusskriterien analog zur AMBASSADOR-Studie gewählt und es wurden insgesamt 709 Patienten eingeschlossen. Geprüft wurde der Einsatz einer adjuvanten Immuntherapie mit Nivolumab 240 mg im 2‑Wochen-Intervall für ein Jahr im Vergleich zu einer verblindeten Placeboinfusion. Die primären Endpunkte waren das krankheitsfreie Überleben in der Gesamtpopulation sowie bei Patienten mit einer positiven PD-L1-Expression mit einem TPS-Score ≥ 1 %. Das mediane krankheitsfreie Überleben in der Gesamtpopulation betrug im Prüfarm 20,8 Monate (95 %-KI: 16,5–27,6), und im Kontrollarm 10,8 Monate (95 %-KI: 8,3–13,9). Die HR betrug 0,70 (98,22 %-KI 0,55–0,90) und es konnte ein statistisch signifikanter Vorteil für eine adjuvante Immuntherapie mit Nivolumab nachgewiesen werden (*p* < 0,001). Besonders deutlich profitierten Patienten mit einem positiven PD-L1-Status: Nach 12 Monaten betrug die DFS-Rate im Prüfarm 67,2 % (95 %-KI: 58,4–74,5) und im Kontrollarm 45,9 % (95 %-KI: 37,1–54,2). Die HR lag bei 0,55 (98,72 %-KI 0,35–0,85) und die Analyse war hochsignifikant (*p* < 0,001). In einer weiteren Subgruppenanalyse zeigte sich zudem ein Vorteil für Patienten, die eine neoadjuvante Cisplatin-haltige Chemotherapie vor radikal chirurgischer Operation erhalten hatten. Die HR für das krankheitsfreie Überleben betrug bei dieser Subgruppe 0,53 (95 %-KI: 0,39–0,72), während bei Patienten ohne neoadjuvante Vortherapie eine HR von 0,91 beschrieben wurde (95 %-KI: 0,69–1,21; [1]). Zusammenfassend ist die CHECKMATE-274-Studie das erste Design mit positiven Ergebnissen für eine Immuntherapie mit Nivolumab im adjuvanten Ansatz für zwei Hochrisikogruppen und erhält von der European Medicines Agency (EMA) die folgende Zulassung: Einsatz einer adjuvanten Immuntherapie mit Nivolumab beim muskelinvasiven Hochrisikourothelkarzinom nach radikaler Resektion bei Patienten mit einem PD-L1-positiven Status (≥ 1 % Tumorzell-PD-L1-Expression). Zur Bewertung der PD-L1-Expression können Tumor Proportion Score (TPS) und Tumor Cell Score (TC-Score) herangezogen werden.

Zusammenfassend liegen aktuell Daten aus 2 Phase-III-Studien zum adjuvanten Einsatz von ICI vor, die ein gemischtes Bild ergeben. Durch eine adjuvante Immuntherapie mit Atezolizumab konnte kein signifikanter Überlebensvorteil erreicht werden. Im Gegensatz dazu zeigte sich durch eine adjuvante Immuntherapie mit Nivolumab ein signifikanter Vorteil des krankheitsfreien Überlebens, insbesondere bei Patienten mit positivem PD-L1-Status. Offen bleibt aktuell, ob dieser Effekt auch auf das Gesamtüberleben übertragbar sein wird, eine kritische Neubewertung sollte daher nach Abschluss der Nachbeobachtung erfolgen.

### Zusammenfassung und Fazit

Neoadjuvante Monotherapien mit Checkpoint-Inhibitoren vor radikaler Zystektomie zeigen keine ausreichende Effektivität, sodass sie nicht als neuer Standard gelten können. Vielversprechender scheinen multimodale Kombinationsregime aus neoadjuvanter Immunchemotherapie, gefolgt von radikaler Zystektomie und adjuvanter Immunmono- oder Immunchemotherapie. Diese „Sandwich“-Regime werden in zahlreichen Phase-III-Studien untersucht, erste Ergebnisse werden für 2023 erwartet.

**Figure 1:**
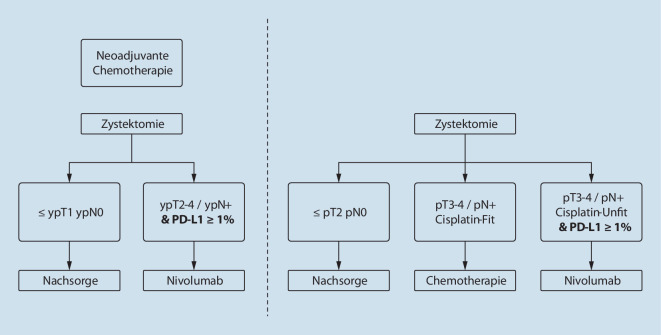

Eine neue Indikation in der uroonkologischen Therapielandschaft ist die adjuvante Immuntherapie mit Nivolumab in zwei Patientengruppen mit einem hohen Rezidivrisiko und PD-L1-positivem Status: Einerseits Cisplatin-ungeeignete Patienten mit einem lokal fortgeschrittenen Tumorstadium und/oder pelviner lymphogener Metastasierung (pT ≥ 3 und/oder pN+) nach erfolgter radikal chirurgischer R0-Resektion eines muskelinvasiven Urothelkarzinoms der Harnblase oder des oberen Harntrakts. Andererseits Patienten nach neoadjuvanter Chemotherapie und radikal chirurgischer Operation, die weiterhin ein muskelinvasives Tumorstadium und/oder eine pelvine lymphogene Metastasierung zeigen (pT ≥ 2 und/oder pN+). Die EMA hat Nivolumab als Adjuvanz für diese zwei Hochrisikogruppen bei PD-L1-positivem Tumorstatus zugelassen. Hieraus ergeben sich neue Therapiesequenzen, die je nach neoadjuvanter Vorbehandlung und Tumorstatus einen neuen perioperativen Standard bedeuten (Abb. 1). Derzeit basiert die Behandlungsstrategie auf den DFS-Daten, sodass eine erneute kritische Bewertung nach Erhalt des Gesamtüberlebens erfolgen sollte.

## Fazit für die Praxis

- Neoadjuvante Monotherapien mit Checkpoint-Inhibitoren vor radikaler Zystektomie können bei unzureichender Effektivität nicht als neuer Therapiestandard gelten.
- Eine neue Zulassung findet sich für den Einsatz einer adjuvanten Immuntherapie mit Nivolumab beim muskelinvasiven Hochrisiko-Urothelkarzinom nach radikaler Resektion und positivem PD-L1-Status („programmed cell death ligand 1“, ≥ 1 % Tumorzell-PD-L1-Expression).
- Aktuelle Studien überprüfen „Sandwich“-Regime aus kombinierter neoadjuvanter Immunchemotherapie, radikaler Zystektomie und adjuvanter Immunmono- bzw. Immunchemotherapie.
